# Biological Agents for Treating Atezolizumab-Induced Psoriasis in Small-Cell Lung Cancer: A Case Report

**DOI:** 10.7759/cureus.61395

**Published:** 2024-05-30

**Authors:** Mototaka Fukui, Yusuke Chihara, Yuki Takahashi, Natsuki Sai, Hiroshi Doi, Yuto Nakakubo, Masahiko Saito

**Affiliations:** 1 Pulmonology, Uji-Tokushukai Medical Center, Uji, JPN; 2 Pulmonology, Kyoto Prefectural University of Medicine, Kyoto, JPN; 3 Immunology, Shiga General Hospital, Moriyama, JPN; 4 Immunology, Kyoto University Hospital, Kyoto, JPN

**Keywords:** secukinumab, risankizumab, atezolizumab, small-cell lung cancer, psoriasis vulgaris

## Abstract

One of the immune-related adverse events from immune checkpoint inhibitors (ICIs) is skin toxicity. Oral corticosteroids are the first-line treatment for severe cutaneous immune-related adverse events. However, corticosteroids may conflict with the efficacy of ICIs. A 55-year-old Japanese man with a history of psoriasis vulgaris was diagnosed with small-cell lung cancer (Stage ⅣA) and administered combined chemoimmunotherapy, including atezolizumab, which resulted in exacerbation of psoriasis. In response, he was treated with biological agents, such as anti-IL-23 and IL-17 antibodies, risankizumab, and secukinumab, respectively, and achieved long-term survival with continued treatment with atezolizumab. This case report suggests that biological agents might be the best course of treatment against autoimmune-related adverse events caused by ICI therapy.

## Introduction

There is an increasing trend of immune checkpoint inhibitor (ICI) usage, improving overall survival not only in patients with lung cancer but also in those with other malignancies [1]. However, ICIs often induce immune-related adverse events (irAEs) that are commonly associated with the activation of immune cells such as T cells, leading to the production of inflammatory cytokines [2]. Therefore, in patients with autoimmune diseases, there is a potential risk of exacerbation of autoimmune symptoms, leading to additional morbidity and increased mortality risk. Psoriasis vulgaris is an autoimmune disease that induces inflammatory keratosis in the skin through immunological reactions and may worsen with ICI therapy. Herein, we present a case of psoriasis vulgaris exacerbation following atezolizumab treatment for small-cell lung cancer. However, ICI therapy was continued using biological agents to control skin symptoms, leading to long-term survival.

## Case presentation

**Figure 1:**
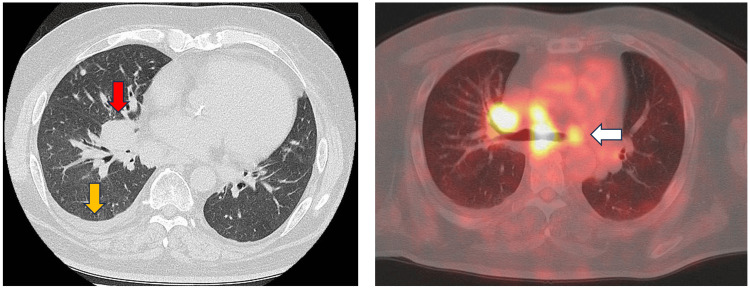
Imaging findings at the initial visit Left image: Computed tomography (CT) scan before first-line chemotherapy showing the right hilar tumor of approximately 4 cm in size (red arrow) and right pleural effusion (yellow arrow). Right image: PET-CT also revealed positive lymph node metastasis in the contralateral mediastinum (white arrow). PET: Positron emission tomography.

A 55-year-old Japanese man was diagnosed with small-cell lung cancer using ultrasound-guided transbronchial needle aspiration of a right lung tumor. Positron emission tomography and computed tomography revealed metastatic lesions in the contralateral mediastinal lymph nodes and right pleural effusion (Figure 1), leading to the diagnosis of cT4N3M1a stage IVA (extensive stage). Treatment with four 21-day cycles of carboplatin (area under the curve 5 mg per mL/min intravenously [IV], day 1) and etoposide (100 mg/m^2^ IV, days 1-3) with atezolizumab (1200 mg IV, day 1) were initiated in January 2020. His medical history included psoriasis vulgaris; however, he did not exhibit any symptoms upon presentation (Table 1).

**Table 1:** Initial blood tests (biochemical) before therapy ProGRP and sIL-2R were elevated, but there were no other major abnormal findings. Alb: Albumin; ALP: Alkaline phosphatase; ALT: Alanine aminotransferase; AST: Aspartate aminotransferase; BUN: Blood urea nitrogen; CPK: Creatine phosphokinase; Cre: Creatinine; CRP: C-reactive protein; Glu: Glucose; Hb: Hemoglobin; LDH: Lactate dehydrogenase; PLT: Platelet; ProGRP: Pro-gastrin-releasing peptide; RBC: Red blood cell; sIL-2R: Soluble interleukin-2 receptor; T-Bil: Total bilirubin; TP: Total protein; WBC: White blood cell.

Biochemical	Reference range	Actual value	Unit
LDH	124-222	259	U/L
AST	13-30	17	U/L
ALT	7-23	16	U/L
ALP	106-322	264	U/L
TP	6.6-8.1	6.2	g/dL
Alb	4.1-5.1	3.4	g/dL
T-Bil	0.4-1.5	0.61	mg/dL
Glu	73-109	174	mg/dL
CPK	41-153	78	U/L
BUN	8-20	15.9	mg/dL
Cre	0.46-0.79	0.78	mg/dL
Na	138-145	139	mEq/L
K	3.6-4.8	4.1	mEq/L
Cl	101-108	105	mEq/L
Ca	8.8-10.1	9.4	mg/dL
CRP	0-0.3	0.09	mg/dL
WBC	33-86	108	10^2^ μL
RBC	435-555	493	10^4^ μL
Hb	13.7-16.8	14.5	g/dL
PLT	15.8-34.8	20.7	10^4^ μL
ProGRP	-81.0	81.5	pg/mL
sIL-2R	122-496	749	U/mL

**Figure 2:**
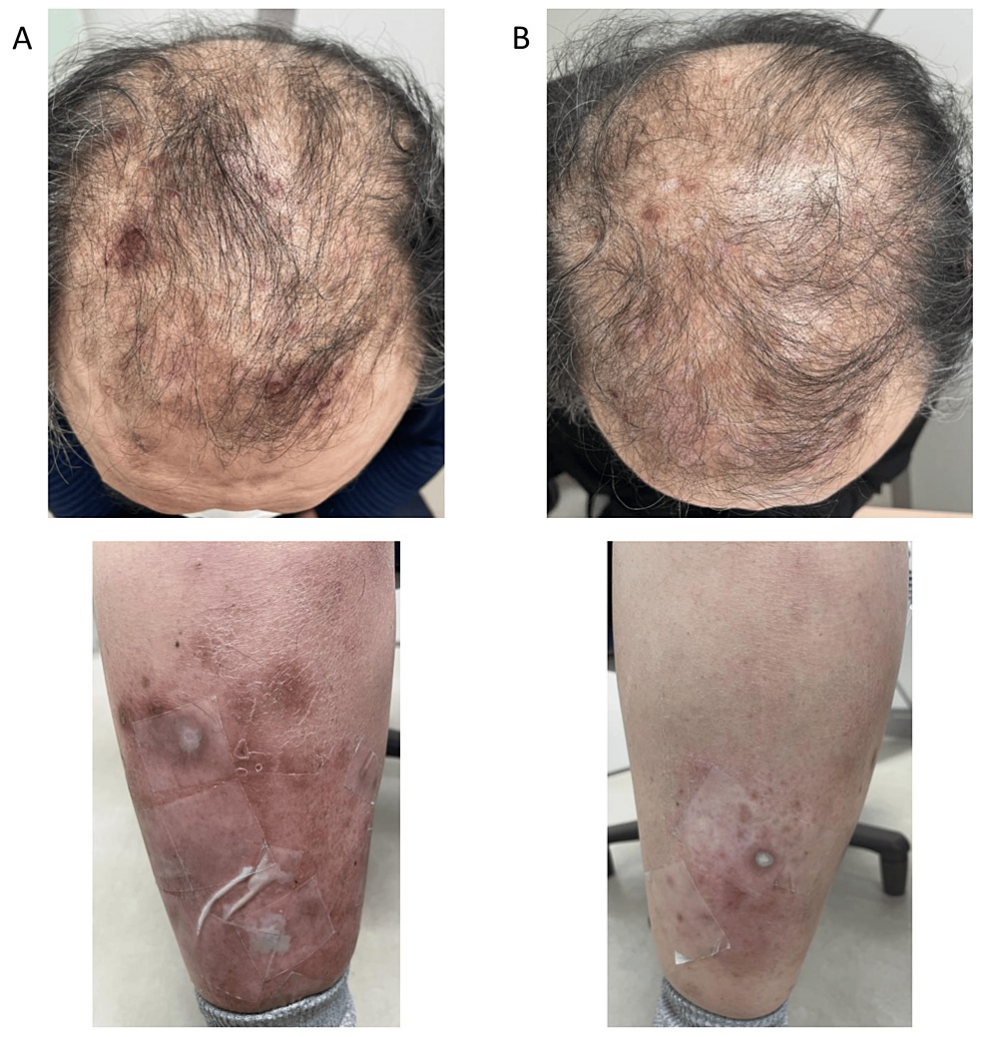
Photos of psoriasis vulgaris before and after treatment with biological agents (A) Before treatment: Erythema on the head and right lower leg. (B) After treatment: Erythema in all areas showed a tendency toward improvement.

The patient achieved complete response (CR) in May 2020 after four courses of initial treatment and started maintenance therapy with atezolizumab in June 2020, which he continued while maintaining CR. However, in July 2020, during the fourth course of maintenance therapy with atezolizumab, he developed erythema on his head, abdomen, and lower extremities (Figure 2, Panel A). He was previously diagnosed with psoriasis vulgaris in 2019 based on a skin biopsy by a dermatologist, and the newly developed erythema shows the same skin lesions present at the time of diagnosis. Hence, he was diagnosed with recurrent psoriasis vulgaris. Topical steroids and antiallergic drugs were administered; as the erythema was classified as grade 2, atezolizumab was continued. However, in March 2021, the erythema worsened to grade 3, and atezolizumab was discontinued during the 13th cycle. He visited the Department of Collagen Disease and was administered an anti-IL-23 antibody (risankizumab; 150 mg subcutaneous injection (SC), day 1, after four weeks, and every 12 weeks). After starting risankizumab, the erythema showed a tendency to improve, and atezolizumab was restarted. However, during the first week of atezolizumab treatment, the erythema worsened and did not disappear completely even after a three-week cycle of risankizumab. At his request, the treatment frequency was changed to every six weeks after 14 cycles, and he was able to continue atezolizumab treatment without further deterioration of the symptoms. In August 2022, the patient was switched to an anti-IL-17 antibody agent (secukinumab; 300 mg SC, day 1, every week from week 1-4, and every four weeks, thereafter), with the expectation of further symptom control, and further improvement in erythema was observed (Figure 2, Panel B).

**Figure 3:**
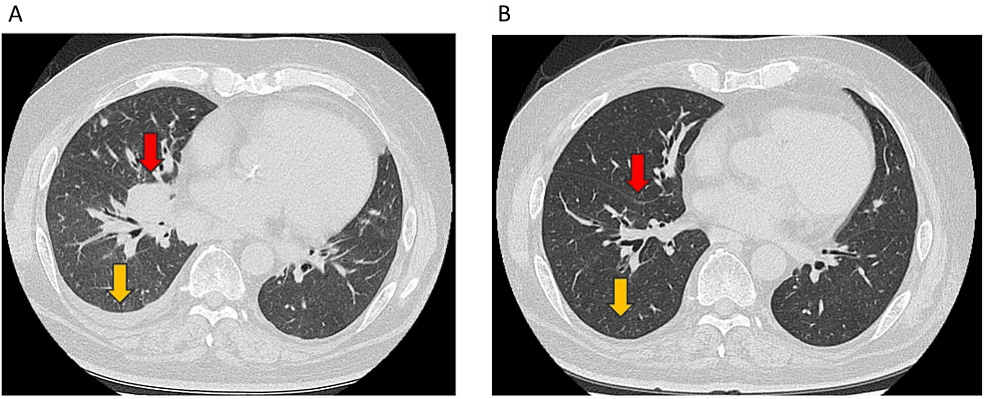
CT scan findings during the patient’s treatment (A) Computed tomography scan findings before first-line chemotherapy showing the right hilar tumor of approximately 4 cm in size (red arrow) and right pleural effusion (yellow arrow). (B) Both the right hilar tumor and right pleural effusion resolved after four courses of maintenance therapy.

Upon recent follow-up, approximately four years after the start of treatment, it was observed that the patient has maintained CR while continuing atezolizumab treatment (Figure 3).

## Discussion

Psoriasis vulgaris is an autoimmune disease that causes inflammatory reactions and epidermal cell proliferation, mainly in the epidermis, owing to immunological abnormalities induced by environmental factors against a background of genetic predisposition toward it [3]. Although cases involving psoriasis vulgaris coinciding with lung cancer are relatively rare, several reports have been published [4,5].

ICIs are one of the most widely used therapies for lung cancer, offering the possibility of long-term survival that is not possible with conventional chemotherapy. Therefore, most patients are treated with ICIs, and combined chemoimmunotherapy has emerged as the standard treatment for small-cell lung cancer as it has been shown to prolong survival. However, patients with pre-existing autoimmune diseases, including psoriasis vulgaris, are at risk of symptom exacerbation, and ICIs are often abandoned.

A meta-analysis of 12 reports (191 cases) on ICI administration in patients with cancer and psoriasis vulgaris [5] revealed that approximately 48% of the patients exhibited exacerbated psoriasis symptoms, although approximately 90% of them were only grade 1 to 2. In addition, the incidence of irAEs other than psoriasis vulgaris did not increase, and the efficacy of ICI therapy was comparable to that in the general population. Regarding lung cancer, case reports are scarce, and some cases have been managed by the administration of topical drugs such as steroids and psoriasis treatment drugs such as PDE4 inhibitors, whereas in others, ICI treatment was terminated because of worsening symptoms [4].

The recommended treatment for cutaneous irAEs includes topical corticosteroids for mild cutaneous irAEs and oral corticosteroids as the primary treatment for moderate or severe cases.

In contrast, treatment guidelines for psoriasis vulgaris state that topical agents, such as steroids, should be applied first. However, if they are not successful or if there are symptoms that impair the patient's quality of life, systemic treatment with immunosuppressive agents or biological agents may be an option. Biological agents are an option when other systemic therapies (e.g., oral immunosuppressive agents and ultraviolet light therapy) cannot be used or are ineffective [6]. There are no differences between available biological agents, and if a biological agent is not sufficiently effective, it can be replaced with another agent [7]. It has been reported that systemic administration of corticosteroids, a nonselective immunosuppressive agent, may interfere with the antitumor effects of ICIs [8], and we believe that a less immunosuppressive treatment is desirable during ICI use, making biological agents a useful option.

A report on the use of biological agents for treating psoriasis symptoms exacerbated by ICIs used secukinumab to control psoriasis exacerbated by pembrolizumab in a patient with malignant melanoma [8]. Similarly, there was a report of psoriasis exacerbated by nivolumab in a patient with malignant melanoma controlled by risankizumab [9]. Pembrolizumab has been reported in advanced non-small-cell lung cancer, in which psoriasis worsened but was controlled with secukinumab and continued without lung cancer recurrence [3]. However, as far as we know, there were no reports involving small-cell lung cancer.

Regarding the usefulness of biological agents in autoimmune diseases other than psoriasis, there is a report of a patient with malignant melanoma complicated by Crohn’s disease who switched from steroids to biological agents before the induction of ICIs and achieved CR by continuing ICIs [10]. It has also been reported that arthritis exacerbated by ICIs can be controlled with biological agents, and ICIs can be continued [11]. Biological agents may help control the exacerbation of other autoimmune diseases by irAEs, not only psoriasis, and allow the continuation of ICI treatment.

Haanen et al. recommended that the use of biological agents before ICI therapy should be considered in patients with autoimmune disease to optimize its effect. Furthermore, patients on steroids should be switched to biological agents to avoid compromising the efficacy of ICIs [12]. The prognosis for small-cell lung cancer is poor, with an average overall survival of only two to four months when untreated and about 10 months with chemotherapy alone [13,14]. However, the IMbrella A, as an extension of the IMpower133 results, demonstrated a five-year survival rate of 12% for combined chemoimmunotherapy (carboplatin, etoposide, and atezolizumab) for small-cell lung cancer [15], suggesting that immunotherapy may result in long-term survival. Therefore, immunotherapy plays an important role in treating patients with small-cell lung cancer. Psoriasis vulgaris is not a fatal disease, and the use of ICIs with biological agents instead of systemic steroids, as in this case, is a valid treatment option for patients with small-cell lung cancer with psoriasis.

## Conclusions

In the present case, recurrence of psoriasis vulgaris occurred six months after the initiation of atezolizumab treatment for small-cell lung cancer. Given the insufficient control with topical medications, including steroids, effective management of psoriasis vulgaris was achieved by employing biological agents, specifically the anti-IL-23 (risankizumab) and anti-IL-17 (secukinumab) antibodies. Consequently, favorable control of psoriasis vulgaris was achieved, and a prolonged response to ICIs was sustained for over four years. Even in patients with psoriasis vulgaris, the concurrent use of biological agents may offer long-term benefits from immunotherapy. The optimal use of ICIs in patients with concomitant autoimmune diseases is yet to be established. Further case reports are expected to contribute to a better understanding of this approach.
